# Natural human *Plasmodium* infections in major *Anopheles* mosquitoes in western Thailand

**DOI:** 10.1186/s13071-016-1295-x

**Published:** 2016-01-13

**Authors:** Patchara Sriwichai, Yudthana Samung, Suchada Sumruayphol, Kirakorn Kiattibutr, Chalermpon Kumpitak, Anon Payakkapol, Jaranit Kaewkungwal, Guiyun Yan, Liwang Cui, Jetsumon Sattabongkot

**Affiliations:** Department of Medical Entomology, Faculty of Tropical Medicine, Mahidol University, Bangkok, 10400 Thailand; Department of Tropical Hygiene, Faculty of Tropical Medicine, Mahidol University, Bangkok, 10400 Thailand; Program in Public Health, University of California, Irvine, CA 92697 USA; Department of Entomology, Pennsylvania State University, University Park, PA 16802 USA; Mahidol Vivax Research Unit, Faculty of Tropical Medicine, Mahidol University, Bangkok, Thailand 10400

**Keywords:** *An. minimus* s.l, *An. maculatus* s.l, *An. annularis* s.l, *An. barbirostris* s.l, Parasite infection, Seasonal dynamics, Malaria transmission

## Abstract

**Background:**

The Thai-Myanmar border is a remaining hotspot for malaria transmission. Malaria transmission in this region continues year-round, with a major peak season in July-August, and a minor peak in October-November. Malaria elimination requires better knowledge of the mosquito community structure, dynamics and vectorial status to support effective vector control.

**Methods:**

Adult *Anopheles* mosquitoes were collected using CDC light traps and cow bait in 7 villages along the Thai-Myanmar border in January 2011 - March 2013. Mosquitoes were determined to species by morphological characters. *Plasmodium*-positivity was determined by circumsporozoite protein ELISA.

**Results:**

The 2986 *Anopheles* mosquitoes collected were assigned to 26 species, with *Anopheles minimus* sensu lato (s.l.) (40.32 %), *An. maculatus* s.l. (21.43 %), *An. annularis* s.l. (14.43 %), *An. kochi* (5.39 %), *An. tessellatus* (5.26 %), and *An. barbirostris* s.l. (3.52 %) being the top six most abundant species. *Plasmodium*-infected mosquitoes were found in 22 positive samples from 2906 pooled samples of abdomens and heads/thoraxes. Four mosquito species were found infected with *Plasmodium*: *An. minimus* s.l., *An. maculatus* s.l., *An. annularis* s.l. and *An. barbirostris* s.l. The infectivity rates of these mosquitoes were 0.76, 0.37, 0.72, and 1.74 %, respectively. Consistent with a change in malaria epidemiology to the predominance of *P. vivax* in this area, 20 of the 22 infected mosquito samples were *P. vivax*-positive. The four potential vector species all displayed apparent seasonality in relative abundance. While *An. minimus* s.l. was collected through the entire year, its abundance peaked in the season immediately after the wet season. In comparison, *An. maculatus* s.l*.* numbers showed a major peak during the wet season. The two potential vector species, *An. annularis* s.l. and *An. barbirostris* s.l., both showed peak abundance during the transition from wet to dry season. Moreover, *An. minimus* s.l. was more abundant in indoor collections, whereas *An. annularis* s.l. and *An. barbirostris* s.l. were more abundant in outdoor collections, suggesting their potential role in outdoor malaria transmission.

**Conclusions:**

This survey confirmed the major vector status of *An. minimus* s.l. and *An. maculatus* s.l. and identified *An. annularis* s.l. and *An. barbirostris* s.l. as additional vectors with potential importance in malaria transmission after the wet season.

## Background

Within the Greater Mekong Subregion (GMS) of Southeast Asia, intensified control efforts have led to a significant reduction in regional malaria disease burden, resulting in major changes in malaria epidemiology. These changes are reflected in the enormous spatial heterogeneity of this area, with transmission hotspots being concentrated along international borders, and the increased prevalence of *Plasmodium vivax*, a parasite more resistant to control measures [1]. In Thailand, most of the malaria incidence has occurred in the western provinces bordering Myanmar, with incidences of 0.55 and 0.46 cases/1000 population in 2013 and 2014. Tak Province has been among the most prevalent provinces for malaria for years, with an incidence rate of 11.7 and 11.67 cases /100,000 population in 2013 and 2014, respectively [2]. In the GMS, there are multiple species of *Anopheles* vectors which present at different seasons or all year round depend on species and locations. Of the many malaria vector species present in the study site, *Anopheles minimus* sensu lato (s.l.) Theobald and *Anopheles maculatus* s.l. Theobald are the main vectors. We found that *An. minimus* s.l. were more abundant during the wet season compared with the dry and hot seasons [3] like the seasonal dynamics in the suspected malaria vector along the Thai-Cambodia border, the *Anopheles barbirostris* group van der Wulp [4]. Continued malaria transmission in Tak is believed to be multifactorial, influenced by ecological, socio-economic, and demographic factors. A better understanding of these factors is deemed crucial for targeted control efforts in the final malaria-elimination phase.

Vector control is one of the most important strategies in malaria control and elimination, and needs to be built on a thorough understanding of vector biology, ecology, behavior, and genetics. For example, recent vector control practices (use of insecticide-impregnated bed nets and indoor residual sprays) might have impacted the feeding behaviors of vectors, resulting in behavioral change in biting time as well as increased propensity for outdoor feeding [5]. In the GMS, malaria vectors are highly diverse in species composition, population dynamics, ecological niche requirements, host feeding preferences and vector competence. Past studies along the Thai-Myanmar border have incriminated three mosquito species complexes - *Anopheles dirus* s.l., *An. minimus* s.l. Peyton & Harrison, and *An. maculatus* s.l. as the most important malaria vectors [6].

Environmental changes associated with anthropogenic land use can cause changes in major vector species community structure, which in turn affects malaria epidemiology. In eastern Thailand, for example, the increasing prevalence of vivax malaria is associated with the replacement of the dominant malaria vector *An. dirus* s.l. by *An. barbirostris* s.l. [4]. This highlights the necessity for continuous monitoring of vector species composition and dynamics in malaria-endemic areas, to facilitate efficient vector control. Although entomological surveys have been conducted on malaria vectors in western Thailand in recent years [3, 7, 8], these studies did not integrate vector abundance with parasite infection to present a more comprehensive picture of the roles of the mosquito species in malaria transmission.

In this study, we surveyed *Anopheles* community structure, seasonal dynamics and *Plasmodium* infections to further illustrate the potential roles of different anopheline species in transmitting human malaria.

## Methods

### Study area

**Fig. 1:**
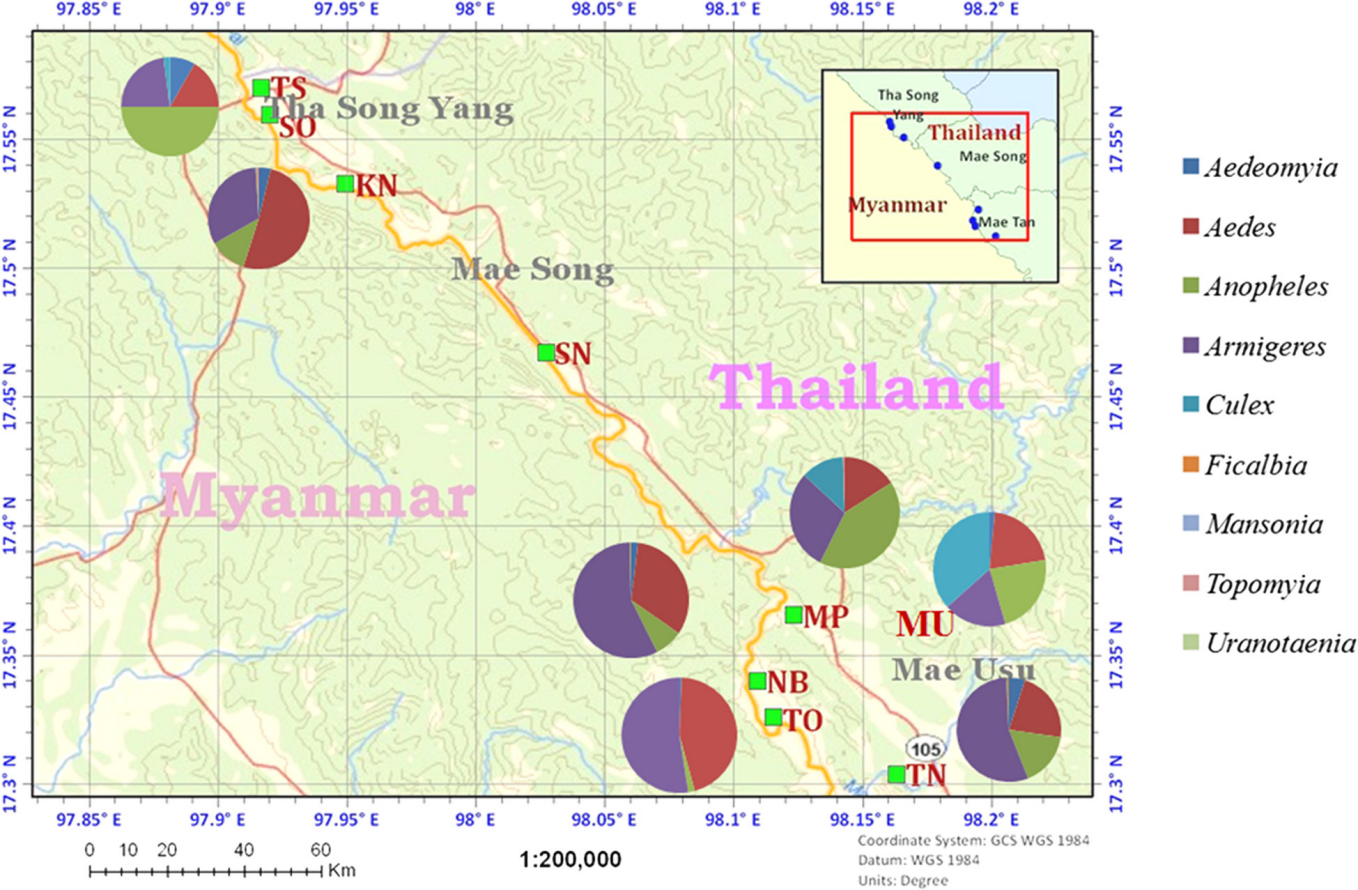
Mosquito collection sites. The seven villages Mae Usu (MU), Tae Nu Ko (TN), Mae Plu (MP), Tha Song Yang (TS), Suan Oi (SO), Tala Oka (TO) and Nong Bua (NB) are along the Thai-Myanmar border. Pie charts represent the abundance of 9 mosquito genera collected in each village

The study sites comprised seven villages - Mae Usu (MU), Tae Nu Ko (TN), Mae Plu (MP), Tha Song Yang (TS), Suan Oi (SO), Tala Oka (TO) and Nong Bua (NB) - all in Thasongyang District, Tak Province, western Thailand, on the Thai-Myanmar border, which is divided by the Moei River (Fig. 1). Records provided by the Bureau of Vector-Borne Diseases showed malaria incidences of 2112, 6247, and 2980 cases from these villages in 2012, 2013, and 2014, respectively. Malaria in this region is seasonal, and typically has two peaks, with the major peak in May-July and the minor peak in October-November [9]. The area consists of 27,166 houses with 137,974 residents, who are mostly farmers. Most houses are near a stream 1–10 m. wide and some swamps (Fig. 1), which are likely mosquito breeding habitats, since mosquito larvae were observed. Monthly data on ambient temperature, rainfall, and humidity, were obtained from the local climatology division (code station 376202), Meteorological Department, Ministry of Information and Communication Technology, Bangkok, Thailand, located in Mae Sod District, Tak Province, about 60 km from the study site.

### Mosquito collections

Adult mosquito surveys were conducted using two methods: CDC light traps and animal baits. Monthly mosquito collection using CDC light traps was performed from March 2012 to March 2013 in three villages (TO, NB and SO) with a total of 30 houses per village. Collection was done for five consecutive nights per month by hanging CDC light traps both indoor and outdoor (20 m away from houses) and with or without CO_2_ attractant for overnight trapping. Surveys were conducted in March-May, June-August, and September-February for a minimum of 15 nights per period, and at least three months per season (dry hot and wet). In addition, in these seven villages we selected 57 houses which have reported malaria cases during 2010–2012. Similarly, CDC light traps with or without CO_2_ were used for indoor and outdoor collections for at least five consecutive nights each month and at least 15 nights per season from January 2011 to March 2013. In the mornings, mosquitoes were removed from the traps. Additional mosquito collection by the cattle bait method was done using 1 cow per night for 2–3 nights per village in MP, SO, and TN in January, April, and May of 2011. In the evening, the cow was tethered inside a net (3.6 × 3.5 × 2 m) with a zippered door on one side. After the cow was removed in the morning, the door was zipped and mosquitoes were collected using an aspirator. Mosquitoes were sorted in the laboratory and species were identified based on morphological characters [10].

### Enzyme-linked immunosorbent assay (ELISA) for *Plasmodium* sporozoite detection

Collected *Anopheles* mosquitoes were kept at −20 °C until detection of malaria parasite sporozoites by ELISA. Field-collected anopheline mosquitoes were first separated into head/thorax (T) and abdomen (A) parts, and examined for circumsporozoite (CS) proteins of *Plasmodium falciparum, P. vivax*-210 (PV210), and *P. vivax*-247 (PV247) [11], either individually or in pools of five to fifteen mosquitoes collected at the same time and location.

### Data analysis

The mean numbers of target mosquitoes collected per season were compared by using the Chi-Square test. Relative abundance of mosquitoes was compared using the Wilcoxon rank-sum test. The level of significance was determined at *P* = 0.05. All data were analyzed using Stata SE package version 13 (Texas, USA).

## Results

### Adult mosquito collection

From January 2011 to March 2013 (for a total of 89 nights) using 26 CDC light traps per night in the seven villages, we collected 6665 adult mosquitoes belonging to 9 genera, among which *Culex* and *Anopheles* mosquitoes were the most abundant (Fig. 1). *Anopheles* mosquitoes were more abundant in the four villages (TS, MP, MU and TK) located closer to the fringe of the mountains. In total, 2986 *Anopheles* mosquitoes were collected (Table 1), giving an average of ~3 *Anopheles* mosquitoes captured per trap per night. The cow bait method collected a total of 99 *Anopheles* mosquitoes. *Anopheles* mosquitoes were assigned to 26 species based on morphology (Table 1). *An. minimus* s.l. was the predominant species, representing 40.32 % of the collected *Anopheles* mosquitoes, followed by *An. maculatus* s.l. (21.43 %) and *Anopheles annularis* s.l. (14.43 %). Our study also revealed considerable differences in mosquito abundance among the villages. Most *Anopheles* mosquitoes were collected in TO (1698), SO (923), and NB (235), whereas 130 were collected in the remaining four villages. In addition, TO and SO also had the greatest species diversity with 21 *Anopheles* species being identified. In each village, *An. minimus* s.l. and *An. maculatus* s.l. were among the most predominant species collected.

**Table 1:** *Anopheles* mosquito species collected in 7 villages of Tak Province, Thailand

Mosquito species	Villages								
	MP	MU	NB	SO	TK	TO	TS	Total	%
*An. minimus* s.l. Theobald	45	24	71	494	8	556	6	1204	40.32
*An. maculatus* s.l. Theobald	2	3	63	273	3	295	1	640	21.43
*An. annularis* s.l. van der Wulp			51	15		365		431	14.43
*An. kochi* Donitz	2	2	24	13	14	106		161	5.39
*An. tessellatus* Theobald			2	3		152		157	5.26
*An. barbirostris* s.l. van der Wulp			6	7	5	87		105	3.52
*An. peditaeniatus* (Leicester)			1	12		50		63	2.11
*An. culicifacies* s.l. Giles			4	33		14		51	1.71
*An. varuna* Iyengar			1	35		5		41	1.37
*An. campestris* s.l. Reid			1	8		13		22	0.74
*An. pseudojamesii* Strickland & Chowdhury					1	19		20	0.67
*An. jamesii* Theobald				9		9		18	0.60
*An. dirus* s.l. Peyton & Harrison	2			6	2	6	1	17	0.57
*An. vagus* Donitz			2	3	6	2		13	0.44
*An. nigerrimus* Giles			1	1		7		9	0.30
*An. philippinensis* Ludlow			2	2		4		8	0.27
*An. indefinitus* (Ludlow)			2	2		3		7	0.23
*An. subpictus* s.l. Grassi		1	1	1		1		4	0.13
*An. dravidicus* Christophers				3				3	0.10
*An. notanandai* Rattanarithikul & Green			1	2				3	0.10
*An. nivipes* s.l. (Theobald)			1			2		3	0.10
*An. aconitus* Donitz		1				1		2	0.07
*An. pseudowillmori* (Theobald)						1		1	0.03
*An. willmori* (James)			1					1	0.03
*An. sawadwongporni* s.l. Rattanarithikul & Green				1				1	0.03
*An. sinensis* Wiedemann	1							1	0.03
Total	52	31	235	923	39	1698	8	2986	

### Seasonal variation

**Fig. 2:**
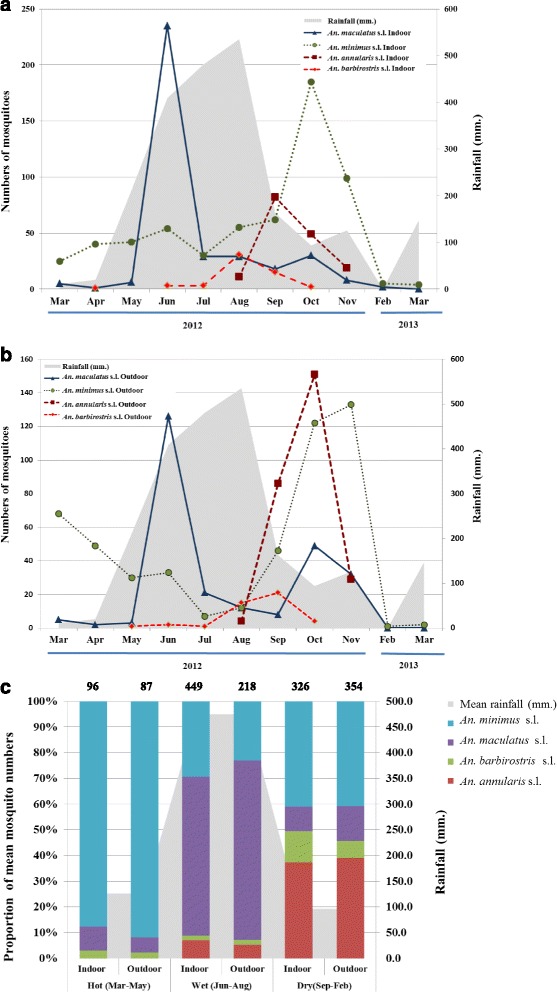
Abundance of four major *Anopheles* mosquitoes. **a** Mosquitoes collected in indoor traps. **b** Mosquitoes collected in outdoor traps. **c** Relative abundance of mosquitoes collected in indoor and outdoor traps during the hot, wet, and dry seasons. Absolute numbers of collected mosquitoes were shown on top of each bar. Shading represents rainfall (mm.) during 2012–2013

Mosquito collection was done in three roughly divided seasons based on rainfall and the temperature variation: hot, wet and dry. All major *Anopheles* mosquito species displayed apparent seasonality (Fig. 2). *An. minimus* s.l. had a peak density during the onset of late wet to dry season (September-November) (Fig. 2a, b). It also had a minor peak in the hot season of March with outdoor collection (Fig. 2b). In comparison, the peak density of *An. maculatus* s.l. was observed in June immediately following the onset of the wet season for both indoor and outdoor collections (Fig. 2a, b). This species had a minor peak in October, which overlaps *An. minimus* s.l. The third most abundant *Anopheles* mosquito, *An. annularis* s.l., was collected in a single period (August-November), overlapping with the peak density of *An. minimus* s.l., particularly at outdoor sites. It is noteworthy that the abundance of *An. barbirostris* s.l. has increased in recent years [Sriwichai P, unpublished data] and this species was collected only in the wet season with a peak density occurring in August and September.

The seasonal dynamics of *Anopheles* mosquitoes were also illustrated by the cumulative abundance of major species, when the three seasons were compared (Fig. 2c). The overall abundance of *An. minimus* s.l.*, An. maculatus* s.l.*, An. annularis* s.l., and *An. barbirostris* s.l. was significantly different between hot and dry seasons (Z = −2.176; *P* <0.05, Wilcoxon rank-sum test). The mean number of *An. maculatus* s.l. collected per season was significantly different between the three seasons (*χ*^*2*^ = 9.773, *df* = 2, *P* < 0.01). Except for *An. minimus* s.l. which was prevalent in all seasons, the other three species were scarce in the hot season. During the wet season, indoor collection was more efficient than outdoor collection for all four species. This was particularly true of *An. maculatus* s.l., the dominant species collected with indoor traps (Fig. 2a, c). In the hot and dry seasons, *An. minimus* s.l. was almost equally abundant in indoor and outdoor traps (Fig. 2c). In dry season, *An. annularis* s.l. was even more abundant than *An. minimus* s.l. Interestingly, the suspected vector *An. barbirostris* s.l. was also relatively abundant in the dry season, and this mosquito appeared to have an indoor preference.

### Malaria infection in collected mosquitoes by ELISA

To identify the specific stages of malaria infection in the collected vectors, in which either oocysts occur in the midgut or sporozoites in the salivary gland, ELISA was performed on abdomens and/or head/thoraxes. All 2936 *Anopheles* mosquitoes collected from light traps and cattle baits were analyzed by sporozoite ELISA to detect malaria parasite infections. Specimens were prepared from 1316 abdomens and 2936 heads/thoraxes. Due to budgetary constraints, only partially pooled abdomens were tested by ELISA, while head/thorax parts from all *Anopheles* mosquitoes were tested to determine infection rate. Twenty mosquitoes were found to be positive for one *Plasmodium* species, whereas two mosquitoes contained mixed *Plasmodium* species infections (Table 2). The infection rates for *An. minimus* s.l., *An, maculatus* s.l., *An. annularis* s.l., and *An, barbirostris* s.l. from total *Anopheles* mosquito samples were 0.76, 0.37, 0.72, and 1.74 %, respectively. Both indoor and outdoor CDC light traps as well as cow baits collected *Plasmodium*-infected mosquitoes. In agreement with observed trends in shifting malaria epidemiology towards *P. vivax* predominance in this region [1], 20 of the 22 infected mosquitoes were positive for *P. vivax*. Consistent with *An. minimus* s.l. and *An. maculatus* s.l. as recognized malaria vector species in western Thailand, thirteen *An. minimus* s.l. and four *An. maculatus* s.l. were *Plasmodium*-positive. In addition, three *An. annularis* s.l. collected from a village in September-October, 2012, were also *Plasmodium*-positive. Of particular note, two *An. barbirostris* s.l. mosquitoes collected with indoor and outdoor traps from two different villages were found to be positive for *P. vivax*. Moreover, 16 of the 22 *Plasmodium-*positive mosquitoes were positive in the head/thorax part, suggesting that these mosquitoes were infective (Table 2).

**Table 2:** *Plasmodium* CS protein positive *Anopheles* mosquitoes (*n* = 22) collected in 7 villages of Tak Province from January 2011 to March 2013

Villages	Collection date	*Anopheles* species	Blood feeding state	Traps	Tested part	Mosquito number	ELISA
MP	Jan-11	*An. minimus* s.l.	Empty	Indoor	A	1	PV210 + PV247
	Jan-11	*An. maculatus* s.l.	Blood fed	Cow bait	A	1	PV247
	Jan-11	*An. minimus* s.l.	Blood fed	Cow bait	T	1	PV247
	Jan-11	*An. minimus* s.l.	Blood fed	Cow bait	A	1	PV247
MU	May-11	*An. maculatus* s.l.	Empty	Indoor	T	1	PV210
NB	Oct-12	*An. maculatus* s.l.	Empty	Indoor	T	1	PF + PV210
	Oct-12	*An. minimus* s.l.	Empty	Indoor	T	1	PV210
	Oct-12	*An. barbirostris* s.l.	Empty	Indoor	T	1	PV210
SO	Aug-11	*An. minimus* s.l.	Blood fed	Indoor	T	1	PV210
	Apr-11	*An. minimus* s.l.	Empty	Outdoor	T	1	PV247
	May-11	*An. minimus* s.l.	Blood fed	Cow bait	A	1	PV247
	Apr-12	*An. minimus* s.l.	Empty	Indoor	T	1	PV210
	Apr-12	*An. minimus* s.l.	Empty	Indoor	A	1	PV210
	Apr-12	*An. minimus* s.l.	Blood fed	Indoor	T	1	PV210
	Jun-12	*An. maculatus* s.l.	Empty	Outdoor	A	1	PF
	Nov-12	*An. minimus* s.l.	Empty	Outdoor	T	8	PV210
TO	Sep-12	*An. annularis* s.l.	Empty	Outdoor	T	1	PF
	Oct-12	*An. annularis* s.l.	Empty	Outdoor	T	1	PF + PV210
	Oct-12	*An. barbirostris* s.l.	Empty	Outdoor	T	1	PV210
	Oct-12	*An. annularis* s.l.	Empty	Outdoor	T	1	PV210
TS	Apr-11	*An. minimus* s.l.	Empty	Indoor	T	1	PV247
	Apr-11	*An. minimus* s.l.	Empty	Indoor	A	1	PV247

*Note*: Adult mosquitoes were dissected into head/thorax (T) and abdomen (A) parts and each part was tested for CS positivity by CS protein ELISA for *P. vivax* (PV210 and PV247) and *P. falciparum* (PF)

*Plasmodium*-positive mosquitoes were found in six of the seven villages surveyed (Table 2). In TO, where *Anopheles* mosquitoes were collected most abundantly, three *An. annularis* s.l. were found infected either singly or with mixed *P. falciparum* and *P. vivax*, while one *An. barbirostris* s.l. was found infected with *P. vivax*. All these infected mosquitoes from TO were also positive in the head/thorax part. From SO, seven *An. minimus* s.l. were found infected with *P. vivax* for both sporozoite types PV210 and PV247, and one *An. maculatus* s.l. was found positive for *P. falciparum*. For the seven positive *An. minimus* s.l., five were ELISA-positive in the head/thorax part. Interestingly, of the four *P. vivax*-positive *An. minimus* s.l. and *An. maculatus* s.l. collected from MP, three were captured from cow baits. It is also noteworthy that even though TS had the lowest *Anopheles* diversity and abundance, two *An. minimus* s.l. collected there were positive for *P. vivax* infection.

In addition to the differences among villages for infected mosquito species, parasite strains also showed geographical variations. In MP and TS, *P. vivax* strain PV247 was exclusively found or was the predominant *P. vivax* strain, whereas in other villages, *P. vivax* strain PV210 was predominant (Table 2). There seemed to be seasonal differences in the prevalence of *P. vivax* strains in our mosquito collections, as PV247 was almost exclusively found from January through May, but not in the rainy season.

### Potential roles of mosquito species in malaria transmission

**Fig. 3:**
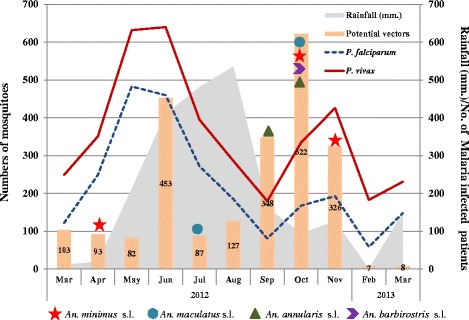
Illustration of monthly cumulative abundance of four major anopheline species (*An. minimus* s.l.*, An. maculatus* s.l.*, An. annularis* s.l.*,* and *An. barbirostris* s.l.), *Plasmodium* positivity, and malaria incidence in the 7 villages. Shading represents rainfall (mm.) during March 2012–March 2013. Star, circle, triangle, and arrow head indicate the *Plasmodium*-positive mosquitoes of the respective species

To explore the potential vectorial status of the *Anopheles* mosquitoes in this region further, we superimposed the malaria incidence data collected from local malaria clinics from March 2012 through March 2013 with the relative abundance each mosquito species as well as *Plasmodium* positivity of the mosquitoes (Fig. 3). There were two peaks of malaria incidence in this region and the major peak was coincident with the highest abundance of the known malaria vectors *An. minimus* s.l. and *An. maculatus* s.l.. During the first peak of malaria incidence, *Plasmodium* infections were identified in these two vector species, further confirming their vectorial status. The minor peak of malaria incidence was from September to November after the wet season, compatible with the presence of all four major *Anopheles* species. Among them, *An. annularis* s.l. was the main species (*n* = 168) and co-dominant with *An. minimus* s.l. (*n* = 108). *Plasmodium* infections were both detected in these two species, pointing to their roles in malaria transmission during the wet-dry season transition.

In addition, all four mosquito species were found malaria parasite positive during the minor peak of malaria incidence, suggesting that they all might be involved in malaria transmission.

## Discussion

Effective vector control as an integrative component of malaria control relies heavily on our understanding of the community structure, seasonal abundance, and infection status of the vector species. Anthropogenic land use activities and insecticide-based control measures have resulted in major shifts of mosquito species abundance and changes in biting behavior, which require continued monitoring of vectors [1]. In this study, we used both CDC light traps (indoor and outdoor) and the cattle bait method to assess the anopheline mosquito abundance in western Thailand in light of the malaria elimination campaign being carried out in this region. This study further revealed the diverse anopheline fauna in this region, confirmed the major vectorial status of several mosquito species, and identified new potential vectors.

Our survey identified 26 *Anopheles* species from a collection of ~3000 adult *Anopheles* mosquitoes. *An. minimus* s.l., *An. maculatus* s.l. and *An. annularis* s.l. are the top three most abundant species, making up more than 75 % of all *Anopheles* species. This finding further supports *An. minimus* s.l. and *An. maculatus* s.l. as the most important malaria vectors in this area [3, 7, 8, 12]. The predominant status of *An. minimus* s.l. as the principal vector in Tak Province is reflected in its absolute abundance (>40 %), which is consistent with observations made 10 years ago [8]. The detection of *P. vivax* CS proteins in both species further demonstrated their competence in transmitting *P. vivax* malaria. In contrast, another important vector *An. dirus* s.l.*,* a forest fringe mosquito, was relatively rare, which is likely due to deforestation.

This study identified *An. annularis* s.l. as a potentially important malaria vector. Normally, *An. annularis* s.l. is considered as zoophagic and predominates in rice fields [13]. It was reported to be susceptible to both PV210 and PV247 *P. vivax* infections in Indonesia [13]. In Bali and Sumba Island, this species occurred at high densities, but did not appear to play an important role in malaria transmission [14]. Climatic and environmental changes might have resulted in habitat changes favoring the breeding of *An. annularis* s.l. in western Thailand, resulting in increased abundance of this species (>14 %) [1, 15, 16]. The detection of both *P. falciparum* and *P. vivax* CS proteins in this species demonstrated its competence in transmitting both parasite species. Moreover, its high density in outdoor traps indicates its potential role in outdoor transmission in the late wet to dry season in this area.

The Barbirostris group is considered a suspected vector of malaria and filariasis in Thailand [4, 17, 18]. *Anopheles barbirostris* s.l. was recognized as a potential vector for *P. falciparum* in Bangladesh and Sri Lanka [19, 20]. Whereas wild-caught *An. campestris-*like mosquitoes were found to be experimentally susceptible to *P. vivax* infection, only one previous report found naturally-caught *An. barbirostris* s.l. to be *P. vivax* positive in Thailand [13]. This study identified *An. barbirostris* s.l. as a relatively abundant species throughout the seasons in both indoor and outdoor collections. Its anthropophilic behavior and PV210 positivity further suggest it as an increasingly important vector for, at least, *P. vivax*. The abundance of newly identified vectors for *P. vivax*, such as *An. barbirostris* s.l., and fewer numbers of other major vectors for malaria in the region, such as *An. dirus* s.l.*,* may contribute to the shift of the prevalence ratio of *P. vivax/P. falciparum* in this area.

Most mosquito species in our study were found seasonally. *An. minimus* s.l. was most abundant during the transition from wet to dry season. In addition, this mosquito species was more abundant in indoor than outdoor collections. In comparison, *An. maculatus* s.l. was the most abundant species in the wet season in both indoor and outdoor collections, consistent with a previous study [8]. Furthermore, confirmation of *Plasmodium*-positivity in this mosquito highlights its role in malaria transmission during the wet season. *An. annularis* s.l. was detected at a similar season as *An. minimus* s.l., suggesting its importance in malaria transmission immediately after the wet season. Further information on each vector’s susceptibility to malaria parasites and their seasonality would be useful for vector control intervention planning which is an important tool to support malaria elimination in the region.

The overall seasonal fluctuation of potential vectors, their *Plasmodium* positivity, and the seasonal dynamics of malaria incidence in the study area are compatible. The region’s apparent transition to *P. vivax* predominance in malaria incidence is also reflected in our detection of mostly *P. vivax* CS proteins in infected vectors. Based on the picture presented in Fig. 3, malaria occurrence in the hot season is probably mediated by *An. minimus* s.l.*,* which is subsequently replaced by *An. maculatus* s.l. in the wet season. After the wet season, the second peak of malaria incidence is likely transmitted by a mixture of competent vector species that prefer either indoor (*An. minimus* s.l.) or outdoor biting (*An. annularis* s.l. and *An. barbirostris* s.l.). While this survey confirmed the major vector status of *An. minimus* s.l. and *An. maculatus* s.l.*,* it revealed two additional species, *An. annularis* s.l. and *An. barbirostris* s.l., as potential vectors after the rainy season. This complex vectorial system of malaria transmission, including seasonality and preferences for either indoor or outdoor feeding, needs to be taken into account when planning for malaria elimination in this region.

## Conclusions

The present study has confirmed that *An. minimus* s.l. and *An. maculatus* s.l. still are the major malaria vectors in the northwestern Thailand. We also identified *An. annularis* s.l. and *An. barbirostris* s.l. as additional vectors which may be important for outdoor malaria transmission after the wet season. The current information can be used to guide vector control program which is an essential tool to support malaria elimination in the region.
